# Enzymatic Synthesis of a Polyol Ester from Levulinic Acid and Trimethylolpropane and Its Tribological Behavior as Potential Biolubricant Basestock

**DOI:** 10.3390/polym12102256

**Published:** 2020-10-01

**Authors:** Wenyuan Zhu, Fangyuan Liang, Hewei Hou, Yuting Chen, Xiaohui Liu, Xudong Zhu

**Affiliations:** 1 Jiangsu Provincial Key Lab of Pulp and Paper Science and Technology, Nanjing Forestry University, Nanjing 210037, China; ppzhuwy12@njfu.edu.cn (W.Z.); fangyuan@njfu.edu.cn (F.L.); hhw99112930@gmail.com (H.H.); cyt33218@njfu.edu.cn (Y.C.); 2 School of Computer Science and Technology, Qilu University of Technology (Shandong Academy of Sciences), Jinan 300175, China; 3 School of Light Industry Science and Engineering, Qilu University of Technology (Shandong Academy of Sciences), Jinan 300175, China; xudongzhu12@outlook.com

**Keywords:** levulinic acid, polyol ester, biolubricant, tribological behaviors

## Abstract

In this study, a polyol ester from levulinic acid (LA) and trimethylolpropane (TMP) was synthesized by enzymatic catalysis in a solvent-free system. The total conversion of TMP reached up to 84% on average after lipase recycling for five times. The produced ester showed excellent lubrication properties, such as high viscosities at 40 °C (86.53 mm^2^/s) and 100 °C (8.91 mm^2^/s), a good viscosity index (49), a low pour point (−27 °C), and a high flash point (223 °C). The frictional wear behavior was evaluated on a four-ball test machine (FTM) by adding the ester into a reference mineral oil. The blend with 10% ester showed a smaller wear scar diameter (WSD) (0.62 mm) when compared with that of pure mineral oil (0.78 mm). The results demonstrated that the obtained ester has huge potential as biolubricant basestock.

## 1. Introduction

Lubricants are widely used in industries, automobiles, aviation machineries, helicopter transmissions, etc., due to their excellent lubrication performances, such as in reducing friction, removing wear particles, increasing efficiency, minimizing energy losses, and distributing heat. It was estimated that the lubricants consumption reached 36.8 million tons around the world in 2019 [1]. Meanwhile, million tons of lubricants enter the environment each year, with more than half ending up polluting the environment through total-loss applications, accidental spillage, non-recoverable usage, volatility, industrial and municipal waste, urban runoff, and refinery process [2,3]. Most of mineral-based lubricants are non-biodegradable and highly toxic to health, water, and environment. Therefore, there is a need to find renewable and biodegradable lubricants as alternatives of mineral products [4]. Biodegradable polyol ester is one of bio-lubricants has being attracted more and more interests because of their excellent lubricity, biodegradability, viscosity-temperature characteristics, and low volatility [5]. So far, the preparation of polyol esters involved transesterification or esterification reaction between polyols concluding pentaerythritol (PE), neopentyl glycol (NPG), and TMP, and various fatty acids, such as oleic acid, linoleic acid, palmitic acid, and octoic acid, etc. However, most of these fatty acids are edible. It is essential to synthesize polyol esters employing non-edible fatty acids.

LA, a five carbon molecule with carboxylic and carbonyl functionalities, can be easily produced from glucose, fructose, starch, and lignocelluloses residues [6]. It has been regarded as one of the most promising twelve molecules derived from biomass. Many LA derivatives have been widely used in the preparation of food flavors, fuels, and composites [7]. Using non-edible LA to replace traditional edible fatty acids for polyols ester preparation is essential. According to a previous study, the esterification between LA and TMP produced a TMP-tri-LA ester, which exhibited excellent lubricant properties as a lubricant basestock. However, the reaction was catalyzed with a less environmentally friendly catalyst, sulphuric acid. Compared with conventional chemical synthesis with mineral acids, enzymatic synthesis showed many advantages, such as high selectivity, environmental friendliness, and good recyclability of catalyst. It is important to achieve environmentally sustainable production of TMP-tri-LA ester using easily recyclable and environmentally friendly bio-enzymes.

In this study, we used an immobilized Candida antarctic lipase B (Novozym^®^435) as catalyst to synthesize TMP-tri-LA ester from LA and TMP. The effects of reaction parameters concluding temperature, enzyme amount, and substrates molar ratio on the synthesis of TMP-tri-LA ester were investigated. The TMP-tri-LA ester was purified with a Rotary Film Molecular Distillation system. The tribological behavior of produced polyol ester was evaluated on a FTM. The reusability of biocatalyst was evaluated. The novelty of the present study is to demonstrate the synthesis of TMP-tri-LA ester via enzymatic catalysis to achieve environmental sustainability.

## 2. Materials and Methods 

### 2.1. Chemicals

TMP was obtained from Fuchen Chemical Co. Ltd. (Tianjin, China); LA with a purity of 99% was purchased from Adamas Reagent Co., Ltd. (Shanghai, China); Immobilized Candida antarctica B (Novozym^®^435) with an initial activity of 10,000 U/g as provided by Beijing Ruisen Co., Ltd. (Beijing, China). Other solvents were obtained from Beijing Chemical Factory (Beijing, China). Mineral oil with a similar viscosity of TMP-tri-LA ester was provided kindly by SINOPEC in Beijing.

### 2.2. Enzymatic Synthesis of TMP-Tri-LA Ester 

The reaction between TMP and LA was preceded in a temperature-controlled rector (Radleys Carousel 6, England). In order to obtain an optimal reaction condition, the temperature was chosen to be 60–90 °C, the molar ratio of LA to TMP was 3.5–6.0, the immobilized lipase amount was 1–4% based on the weight of substrates. The pressure was maintained constantly at 100 Mbar to remove the generated water during esterification. After one reaction cycle of 72 h, the immobilized lipase was separated from the reaction mixture with a glass filter (grade 160) and washed with 2-propanol [8]. New substrates were fed in reactor for next reaction cycle.

### 2.3. Analytical Procedure

Samples (15 μL) were taken and dissolved in ethyl acetate of 1.5 mL. The concentrations of substrates were quantified using a gas chromatography (GC) (Shimadzu, GC-2010, Kyoto, Japan) equipped with a DB-1ht capillary column (30 m × 0.25 mm × 0.1 μm; J and W Scientific, Folson, CA, USA) and a flame ionizing detector (FID). The column temperatures were increased to 132, 180, and 300 °C with increments of 12, 30, and 20 °C/min, respectively. The temperatures of injector and detector were set at 300 °C. The conversion rate of TMP was calculated by following Equation (1). Each measurement had 3 replications,

Conversion rate of TMP (%) = (C_0_−C_T_)/C_0_ × 100% (1)

where, C_T_ and C_0_ were the final and initial concentrations of TMP in sample, respectively.

The content of polyol esters was calculated by Equation (2),

The content of polyol ester = S/S_t_ × 100% (2)

where S was the integral area of individual ester, such as monoester, diester, and trisubstituted ester and S_t_ was total integral area of three esters.

Mass spectra were recorded on a VG Auto Spec-M (Manchester, UK).1. H NMR spectra were obtained using a Bruker AR X 400 Spectrometer (400 MHz, Karlsruhe, Germany).

### 2.4. TMP-tri-LA Ester Purification

The reaction mixture was distilled with a Rotary Film Molecular Distillation system (VTA GMBH and Co. KG, Niederwinkling, Germany) at 140 °C and 1 Mbar to remove excessive LA. After that, the crude triester obtained from first distillation was further distilled at 230 °C and 0.009 Mbar to get the final TMP-tri-LA ester with a purity of 95% above.

### 2.5. Viscosity of TMP-tri-LA Ester

Kinetic viscosity is an important characteristic to exhibit the relationship between viscosity and temperature. The property of viscosity–temperature directly represents the lubricant performance and determines its application. Viscosity measurements were conducted at 40 and 100 °C using Cannon Fenske viscometer tubes in a Cannon Constant Temperature Viscosity Bath (Hunan Petrochemical Instrument Co., Ltd., Changsha, China). Viscosity and viscosity index were calculated according to ASTM methods D445, and D2270, respectively.

### 2.6. Pour Points of Tmp-Tri-La Ester

Pour point is another key parameter to assess the performance and application of a potential lubricant. Pour points were determined according to an ASTM method D97 with an accuracy of ±3 °C on an Automatic Pour Point Tester (Hunan Petrochemical Instrument Co., Ltd., Changsha, China). The temperature of pour point was recorded when liquor flow stopped.

### 2.7. Flash Point of TMP-Tri-LA Ester

Flash point was defined as the minimum temperature that ignited volatiles. The lower flash point of poyol ester has, the higher fire hazard occurs. Flash point was determined using a Flash Point Tester (Hunan Petrochemical Instrument Co., Ltd., Changsha, China) according to an ASTM method of D93. The temperature which causes the vapor above the surface of the liquid to be ignited is the temperature of flash point at ambient barometric pressure.

### 2.8. Tribological Behavior 

Wear tests were determined using a FTM (Hunan Petrochemical Instrument Co., LTD., Changsha, China) according to ASTM D4172 method.

## 3. Results and Discussion

### 3.1. Reaction Conditions Optimization

**Figure 1:**
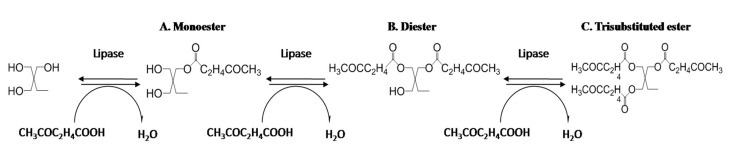
Schematic representation of esterification reaction between LA and TMP.

Figure 1 shows the schematic representation of enzymatic synthesis of TMP-tri-LA ester from LA and TMP. The reaction was proceeded in a solvent free system to avoid the release of toxic solvents.

**Figure 2:**
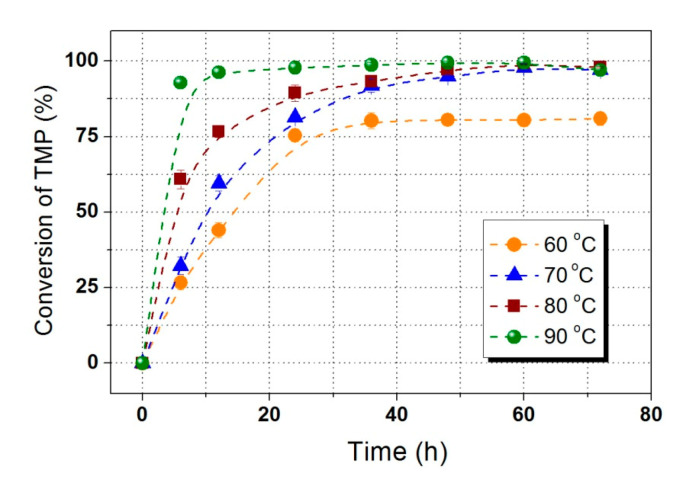
Effects of reaction temperature on the synthesis of TMP-tri-LA ester.

**Figure 3:**
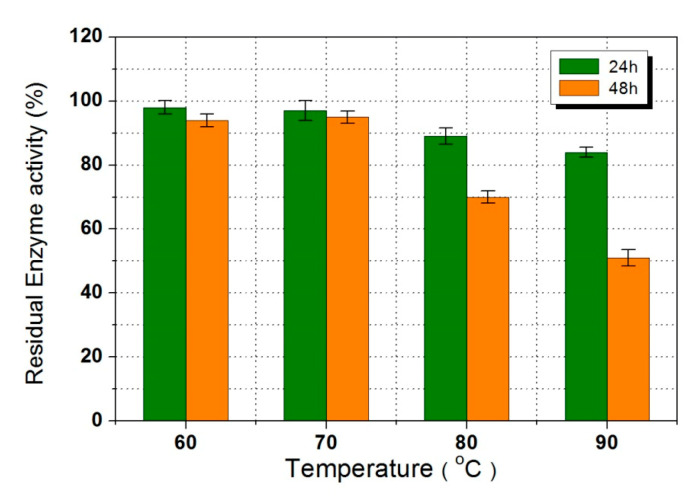
Residual enzymatic activity after 24 and 48 h of reaction at different temperatures.

Temperature usually plays an important role in process of enzymatic catalysis. Increasing temperature facilitates reaction rate due to the improvement of interphase mass transfer, the conformational flexibility of enzymes, and the release of water from the system. Figure 2 shows the profile of TMP conversion at 60, 70, 80, and 90 °C with a substrates molar ratio of 5:1 (LA to TMP) and an enzyme loading of 4%. The conversion of TMP reached to 92.85% at 90 °C after 6 h, but only 32.22% was observed at 70 °C with same reaction time. After 72 h, higher TMP conversions of 99.43% and 97.88% were obtained at 90, and 70 °C, respectively. Obviously, the high temperature enhanced the formation of trisubstituted TMP esters. However, too high temperature caused enzyme denaturation resulting in a decrease of residual enzyme activity of lipase. Figure 3 displays the residual enzymatic activity after 24 and 48 h of reaction at different temperatures, a significant decrease of catalytic efficiency was found. The lipase maintained 94.05% (24 h) and 95.11% (48 h) of its original activity at 70 °C. When the temperature increased to 80 °C, the residual enzymatic activity decreased to 76.31% (24 h) and 59.72% (48 h). Therefore, 70 °C was proposed to be the optimum reaction temperature for effective synthesis of TMP-tri-LA ester. The results were similar to the study reported by Åkermana et al. [8].

**Figure 4:**
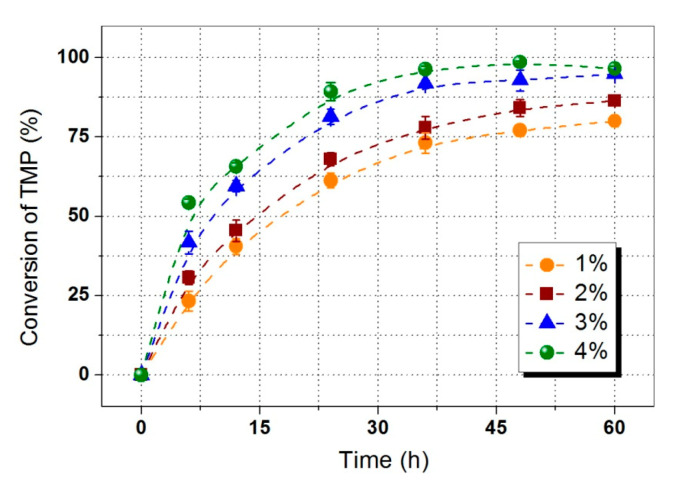
Influence of enzyme amount for TMP-tri-LA ester synthesis.

In order to find the optimal amount of lipase used in the esterification reaction, the effect of enzyme amount from 1–4% based on substrates weight on TMP conversion was investigated as demonstrated in Figure 4. 86.38% of total TMP conversion was achieved after 60 h with an enzyme amount of 2%. Increasing the lipase amount to 3% significantly improved the synthesis of TMP-tri-LA ester. When the enzyme amount was further increased to 4%, no reaction conversion enhancement was found, which could be explained by the fact that mass transfer and the access of active sites of enzyme were limited under high enzyme loading [9]. Therefore, an optimal enzyme amount of 3% based on the weight of substrates was suggested.

**Figure 5:**
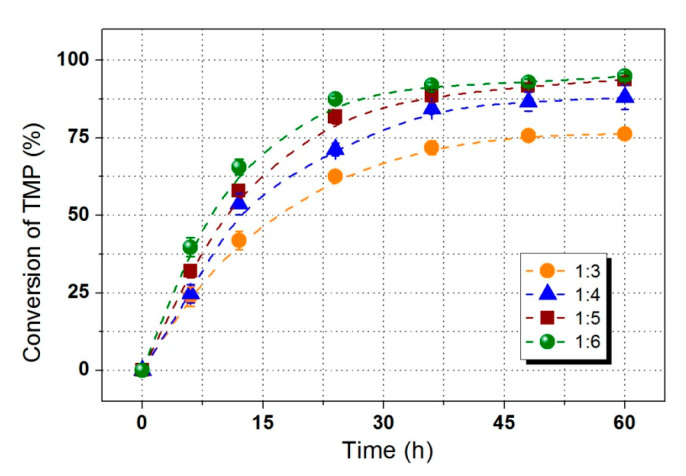
Influence of substrates molar ratio on the synthesis of TMP-tri-LA ester.

**Figure 6:**
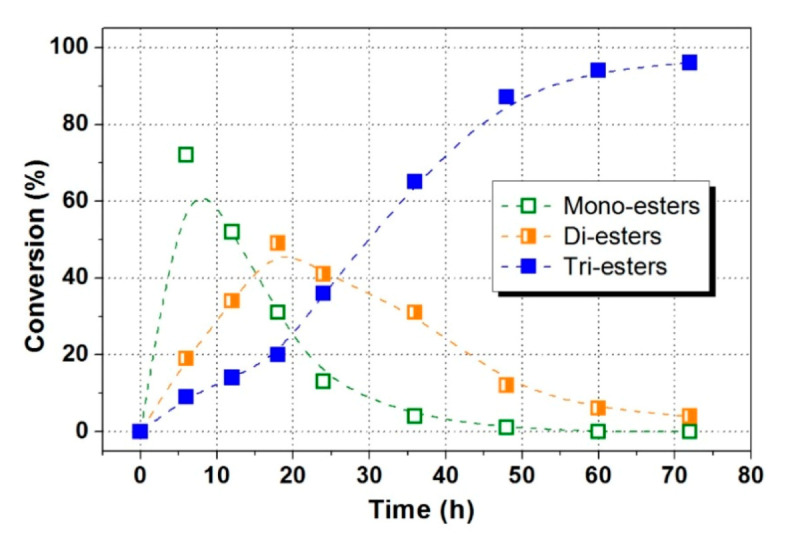
The formation of mono-, di-, and tri-esters during enzymatic esterification.

The effect of molar ratio of initial substrates on TMP-tri-LA ester synthesis was analyzed at different ratios of 3:1–6:1 (LA:TMP) at 70 °C applying 3% enzyme loading as presents in Figure 5. High molar ratio of substance significantly enhanced the reaction speed. A high conversion of TMP (88.00%) was observed at molar ratio of 1:4 (TMP to acid). It is found that the LA to TMP ratio mainly influenced the product composition in terms of the relative concentration of mono-substituted esters (mono-esters), di-substituted esters (di-esters), and tri-substituted ester (tri-esters), which was similar with the results from a previous study [8]. Although, employing a higher molar ratio (1:6) further increased TMP conversion, the removal of excessive LA will increase the purification cost of TMP-tri-LA ester. Therefore, an optimal ratio of 4:1 was suggested for TMP-tri-LA ester synthesis. Figure 6 shows the formation of mono-esters, di-esters, and tri-esters during enzymatic esterification under optimal conditions. The content of tri-esters in reaction mixture reached to 96%.

### 3.2. Biocatalyst Recycling

**Figure 7:**
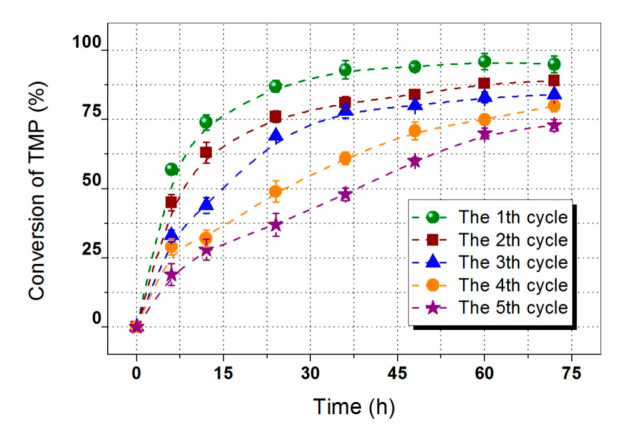
Reusability of biocatalyst during the synthesis of TMP-tri-LA ester.

Compared with conventional chemical catalysts, biocatalysts exhibit many advantages, such as high selectivity and environmental friendliness. However, a relatively high price usually limits its industrial application. Recyclable immobilized biocatalyst can significantly reduce catalytic cost. Figure 7 shows the reusability of biocatalyst during the synthesis of TMP-tri-LA ester. The total conversion of TMP in the first and the second cycles were 95.25% and 89.87%, respectively. Due to enzyme denaturation and/or leakage from the carrier, the residual activity of lipase decreased. Similar results were found in a previous study [10]. Therefore, the conversion of TMP in fourth and fifth cycle decreased to 80.19%, and 73.07% after 72 h, respectively. Biocatalyst stability test showed that the lipase remained a high activity and the total conversion of TMP was above 84% on average after 72 h within 5 cycles.

### 3.3. Physico-Chemical Properties of TMP-Tri-LA Ester

The crude product was purified using a Rotary Film Molecular Distillation system to get the final TMP-tri-LA ester with a purity of above 95%. ^1^H NMR spectra of trisubstituted TMP ester were obtained as follow: ^1^H NMR (CDCl_3_, δ ppm): 0.89–0.92 [s, 3H, (–CH_3_)], 1.48–1.50 [s, 2H, (–CH_2_–CH_3_)], 2.20 [s, 9H, 3 × (–CO–CH_3_)], 2.58–2.60 [s, 6H, 3×(–CO–CH_2_–)], 2.76–2.79 [s, 6H, 3 × (–CH_2_–CO–)], 4.03 [s, 6H, 3 × (–CO–CH_2_–)].

From Table 1, the TMP-tri-LA ester exhibited a very low pour point (−27 °C). Compared with other fat acid-based polyol esters in recent researches, the LA-based TMP polyol ester exhibited a competitive low temperature characteristic [11,12]. The low pour point of −27 °C was equal to that of product from castor oil and TMP (−27 °C) [11] and lower than that of the polyol ester (−18 °C) from rapeseed oil and TMP, and product (pour point −6 °C) from rubber seed oil and TMP [12]. The viscosity index was very high, up to 49. The temperature of flash point was defined as the minimum temperature that ignited volatiles. The lower flash point of polyol ester has, the higher fire hazard occurs. The flashpoint of TMP-tri-LA ester was determined to be 223 °C above, which reached the standard of low evaporation requirement of lubricants. Generally, polyol esters with a flash point ≥165 °C can lie well within the range of hydraulic oils [13].

### 3.4. Tribological Behaviors of TMP-Tri-LA Ester

**Figure 8:**
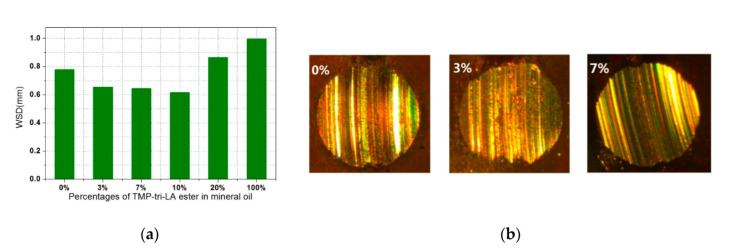
(**a**) WSDs of reference mineral oil containing different percentages of TMP-tri-LA ester. (**b**) WSDs microscope of reference mineral oil containing different percentages of TMP-tri-LA ester.

Tribological behaviors were investigated on a FTM by adding different percentages (0%, 3%, 7%, 10%, 20% and 100%) of TMP-tri-LA ester into a reference mineral oil. Figure 8 shows the WSD results. When TMP-tri-LA ester addition increased from 0% to 10%, the WSD became smaller generally. The smallest WSD was found to be 0.62 mm with 10% addition, which can be attributed to the increase in molecular binding due to a large number of ester groups in the mixture. However, a higher percentage of addition showed non-remarkable improvement in reducing friction. 100% of TMP-tri-LA ester displayed the largest WSD of 0.99 mm. The increase of WSD can be explained by the rubbing surface of metal test panel becoming corroded by LA in the ester or the pure ester, which could not form more stable lubricant film. This phenomenon was similar to that of palm oil-based TMP ester [14]. The results above showed that the produced TMP-tri-LA ester could reduce wear efficiently as a biolubricant stock. Fernandez et al. found that TMP ester could be acted as a wear reducer when it was added into polyalphaolefin with a low viscosity [15]. Figure 8 shows the microscope images of wear scar with different percentages of TMP-tri-LA ester in mineral oil. All the wear scars were circular in shape, which meant all the samples exhibited excellent lubricity. Although, the edge of the wear scars was slightly ragged and the worn surface showed abrasive wear after adding ester into mineral oil, and no severe adhesive wear was found. The light spot on the worn surface indicated that the direct metal-to-metal contact occurred. As shown in these microscope images, the light area minified slightly with the addition of TMP-tri-LA ester into mineral oil. Obviously, the samples with 10% ester significantly reduce the direct metal-to-metal contact and generated the lowest WSD of 0.62 mm. It was believed that this mixture sample could successfully create a protective film. For pure TMP-tri-LA ester (100%), the edge was slightly ragged and polished by metal particle, and its WSD became larger. This increase of WSDs and light spot was also caused by the oxygen corrosion of TMP-tri-LA ester. The tribochemical reaction was occurred on the metal surface resulting in metal corrosion. Masabumi et al. investigated the tribochemical wear using smooth and flat surface and found the partial tribochemical reaction between metal material and lubricant. The reaction had a negative effect on tribological behaviors of biolubricant [16]. The above results showed that the TMP-tri-LA ester has huge application potential in biolubricant preparation.

## 4. Conclusions

LA is a promising chemical derived from biomass. In this study, a new polyol ester from LA and TMP was prepared with immobilized lipase *Candida antarctica* as catalyst in a solvent free system. The obtained ester exhibited superior properties of lubricant, such as high viscosity index, low pour point, and high flash point. Tribological behaviors were investigated on a FTM by adding different percentages of TMP-tri-LA ester into reference mineral oil. A smaller WSD was found to be 0.62 mm for 10% addition compared to that of pure mineral oil. It suggested that the produced ester could be regarded as a potential base stock for production of high performance lubricant.

## Figures and Tables

**Table 1:** Physico-chemical properties of LA-based polyol ester.

Property	Value
Viscosity at 40 °C, (mm^2^/s)	86.53
Viscosity at 100 °C, (mm^2^/s)	8.91
Viscosity index	49
Pour point (°C)	−27
Flash point (°C)	223
